# Blockade of LAG-3 in PD-L1-Deficient Mice Enhances Clearance of Blood Stage Malaria Independent of Humoral Responses

**DOI:** 10.3389/fimmu.2020.576743

**Published:** 2021-01-14

**Authors:** Raquel Furtado, Laurent Chorro, Natalie Zimmerman, Erik Guillen, Emily Spaulding, Shu Shien Chin, Johanna P. Daily, Grégoire Lauvau

**Affiliations:** ^1^Department of Microbiology and Immunology, Albert Einstein College of Medicine, Bronx, NY, United States; ^2^Department of Medicine, Albert Einstein College of Medicine, Bronx, NY, United States

**Keywords:** checkpoint therapeutic blockade, PD-1/PD-L1/LAG-3, inhibitory receptors, malaria, humoral immunity, LAG-3+ CD4+ and CD8+ T cells, PD-L1 and PD-1 knockout mice, *Plasmodium yoelii*

## Abstract

T cells expressing high levels of inhibitory receptors such as PD-1 and LAG-3 are a hallmark of chronic infections and cancer. Checkpoint blockade therapies targeting these receptors have been largely validated as promising strategies to restore exhausted T cell functions and clearance of chronic infections and tumors. The inability to develop long-term natural immunity in malaria-infected patients has been proposed to be at least partially accounted for by sustained expression of high levels of inhibitory receptors on T and B lymphocytes. While blockade or lack of PD-1/PD-L1 and/or LAG-3 was reported to promote better clearance of *Plasmodium* parasites in various mouse models, how exactly blockade of these pathways contributes to enhanced protection is not known. Herein, using the mouse model of non-lethal *P. yoelii (Py)* infection, we reveal that the kinetics of blood parasitemia as well as CD4^+^ T follicular helper (T_FH_) and germinal center (GC) B cell responses are indistinguishable between PD-1^-/-^, PD-L1^-/-^ and WT mice. Yet, we also report that monoclonal antibody (mAb) blockade of LAG-3 in PD-L1^-/-^ mice promotes accelerated control of blood parasite growth and clearance, consistent with prior therapeutic blockade experiments. However, neither CD4^+^ T_FH_ and GC B cell responses, nor parasite-specific Ab serum titers and capacity to transfer protection differed. We also found that i) the majority of LAG-3^+^ cells are T cells, ii) selective depletion of CD4^+^ but not CD8^+^ T cells prevents anti-LAG-3-mediated protection, and iii) production of effector cytokines by CD4^+^ T cells is increased in anti-LAG-3-treated versus control mice. Thus, taken together, these results are consistent with a model in which blockade and/or deficiency of PD-L1 and LAG-3 on parasite-specific CD4^+^ T cells unleashes their ability to effectively clear blood parasites, independently from humoral responses.

## Introduction

The remarkable success of anti-cancer checkpoint blockade therapies have provided formal proof of concept that reinvigorating T cells with their effector potential is a feasible and realistic approach to achieve more effective immune-based therapies of human diseases (1). Understanding signals that can modulate and shape T cell functions has established the importance of costimulatory and coinhibitory pathways in T cell biology (2). While co-stimulation is essential to prime fully functional T cells, expression of inhibitory receptors such as program cell death 1 (PD-1) and Lymphocyte Activation Gene 3 (LAG-3) (3) expressed on activated T cells, enables regulation and optimal memory formation. Expression of PD-1 facilitates the down regulation of activated T cell functions through interactions with its major ligands PD-L1 and PD-L2. LAG-3 is a CD4 homolog that binds MHC class II on antigen-presenting cells with higher affinity than CD4 and can act as a negative regulator of T cell functions. Recently, the fibrinogen like protein 1 (FGL-1) that is naturally expressed by liver cells and overexpressed by some tumor cells, was also characterized as a new ligand for LAG-3 which can potently prevent T cell activation upon binding (4).

In the context of chronic viral infections (HIV, Hepatitis C) and in tumors, sustained antigenic stimulation and an immuno-suppressive microenvironment promote the onset of T cells that maintain high levels of PD-1 and LAG-3 and exhibit an “exhausted” or dysfunctional phenotype, which prevents robust host protective effector T cell responses (1, 5). Blockade of the PD-1/PD-L1 pathway can -at least partially- rescue T cell functionality and allow for virus and tumor clearance (6). The fundamental importance of targeting T cell inhibitory pathways for therapeutic purposes has been highlighted in various cancers (1). More recently, several reports have also provided strong evidence that these inhibitory pathways may account for the lack of long-term sterilizing immunity in malaria-infected patients (7). Expression of high levels of PD-1 and LAG-3 on CD4^+^ and CD8^+^ T cells, and expansion of such cells among peripheral blood mononuclear cells (PBMCs) of malaria-infected patients has been reported (8, 9). In addition, transcripts encoding the butyrophilin family member butyrophilin-like 2 (BTNL2), another negative regulator of T-cell activation, were increased during malaria infection compared to convalescent uninfected controls (10). Likewise, a subset of “atypical” memory B cells (CD21^-^CD27^-^CD10^-^), originally described as “exhausted” B cells in HIV- and HCV-infected patients (11, 12), are expanded in malaria patients and may contribute to the lack of effective long-lived immunity against this parasite (9, 11). Both PD-1 and LAG-3 inhibitory molecules are also upregulated in various surrogate non-lethal mouse models of blood stage malaria (8, 12, 13), and, as in chronic viral infections and tumors, their selective blockade accelerates pathogen elimination. While enhanced parasite clearance is accounted for by improved CD4^+^ T and B cell/antibody responses (8), other mechanisms involving the restoration of CD8^+^ T cell cytolytic functions are also proposed (13).

Because of the potential clinical significance of these inhibitory pathways during malaria infection, we investigated the relative contribution of PD-1/PD-L1, LAG-3 or both pathways in the clearance of *Plasmodium* parasites from the blood of infected mice, and the modulation of host immune responses to improve infection outcomes. Using the non-lethal and non-chronic *P. yoelii (17XNL1.1)* mouse model of blood stage malaria infection, we report that mice genetically deficient for PD-L1 and PD-1 exhibit comparable kinetics of blood parasitemia to WT counterparts. We also found that LAG-3 blockade in PD-L1^-/-^ mice accelerates parasite clearance as in WT mice co-treated with anti-LAG-3/PD-L1 monoclonal Abs (mAbs). Yet, while therapeutic blockade of PD-L1/LAG-3 in WT mice promotes a greater magnitude of CD4^+^ T_FH_ and GC B cell responses, that of LAG-3 in PD-L1^-/-^ mice fails to enhance these responses. Rather, we reveal that blockade of LAG-3, which is mostly detected on activated CD4^+^ and CD8^+^ T cells, promotes parasite clearance independent of parasite-specific Abs and CD8^+^ T cell responses. Since CD4^+^ T cells are required for blood parasite elimination, these results are consistent with a model in which blockade of LAG-3 and PD-L1 act synergistically on CD4^+^ T cells to mediate direct parasite clearance.

## Materials and Methods

### Mice

*This study was carried out in strict accordance with the recommendations by the animal use committee at the Albert Einstein College of Medicine that approved the animal protocol*.

Wild-type (WT) C57BL/6J (B6) 6-12 week-old male mice, PD-1*^-/-^* and PD-L1^-/-^ (Gift Stan Nathenson, Einstein) were housed and bred in our SPF animal facility for all experiments.

### *Plasmodium* Infections, Blood Parasitemia, and Adoptive Serum Transfers

#### Infections

*Plasmodium yoelii (Py) 17XNL(1.1)* parasites (stock MRA-593) and GFP expressing parasites (stock MRA-817) were obtained from the Malaria Research and Reference Reagent Resource Center as part of the BEI Resources Repository (NIAID, NIH, Manassas, VA. The *P. yoelii*, strain *17XNL(1.1)*, MRA-593 was contributed by D. J. Carucci. *Py*-infected red blood cells (iRBC) from a frozen stock (−80°C in Alsever’s solution, 10% glycerol) were intraperitoneally (i.p.) injected into a WT B6 mouse and grown for ~4 days. When parasitemia reached 2%–5%, 2x10^5^ *Py* iRBCs were injected intravenously (i.v.) into each experimental mouse.

#### Parasitemia and Weight

Blood parasitemia was determined by flow cytometry on 1 µl of blood obtained by cutting the tip of the mouse tail with a sterile razor. Blood was fixed in 200 µl of 0.025% glutaraldehyde in PBS 1mM EDTA before washing and permeabilization with 0.25% Triton X-100 in PBS for 5 min. After centrifugation, RBCs were incubated in 1mg/ml RNAse A (Sigma) for 30 min at room temperature (RT) and stained with 0.5 µM of the YOYO-1 dye (Invitrogen) for 30 min at RT and directly analyzed on a BD FACSCanto II (Becton Dickinson, CA). RBCs were gated based on forward and side scatter, and parasitemia was determined as the frequency of YOYO-1^+^ cells among all gated RBCs. Manual counting of Giemsa-stained blood smears by microscopy gave comparable parasitemia results. Mice were individually weighed (recorded in grams) on a tared weighing scale prior to blood collection.

#### Serum Transfer Experiments

Serum harvested from blood collected by cardiac puncture from indicated mice, at indicated time points was stored at -80°C. Serum was thawed, pooled within treatment groups and 150 µl of pooled serum were transferred to experimental mice intravenously (i.v.) prior to infection with *P. yoelii 17XNL(1.1)*. Pooled serum was heat inactivated at 56°C for 30 min prior to use in ELISAs.

### Antibody Blockade and Depletion

#### PD-L1, PD-1, and LAG-3 Blockade

200 µg, unless otherwise indicated in figure legends, of polyclonal rat IgG (BioXcell; isotype) or anti-LAG-3 (clone C9B7W, BioXcell) and/or anti-PD-L1 (10F.9G2, BioXcell) or anti-PD-1 (RMP1-14, BioXcell) blocking Abs were injected i.v. into blood-stage parasitized mice beginning on day 9 or 12 after infection (as indicated) and every 3 days until day 15 or 18 as indicated. Antibodies which contained endotoxin levels above 3 EU/ml (Kinetic-QCL Kinetic Chromogenic LAL Assay, Lonza) were depleted of endotoxin with the ToxinEraser Endotoxin Removal kit (GenScript) following manufacturer’s instructions.

#### T Cell Depletions

150µg of anti-CD4 (clone GK1.5) or anti-CD8β (H35) mAbs were injected intraperitoneally (i.p.) one day before *P. yoelii 17XNL(1.1)* infection, and then at day 4 and 9 post infection.

### Preparation of Cell Suspensions for Flow-Cytometry (FACS) Analysis

Spleens were dissociated on nylon meshes (100µm) and incubated at 37°C for 20 min in HBSS medium containing 4,000 U/ml of collagenase I (Gibco) and 0.1 mg/ml of DNase I (Roche), and RBCs further lysed with 0.83% NH_4_Cl buffer. Cells were resuspended in FACS buffer (PBS 1% FCS, 2mM EDTA, 0.02% sodium azide) and used for the different analyses detailed below.

### Cell Staining for FACS Analysis

Cell suspensions were incubated with 2.4G2 antibody for 15 min at 4°C and further stained with various antibody cocktails (**Supplementary Table 1**) in FACS buffer. For detection of intracellular IFNγ, TFNα and IL-2 staining, cells were incubated for 3–4 h at 37°C, 5% CO_2_ in RPMI 1640 (Invitrogen) 5% FCS, Golgi Plug (BD, 1/1,000), Golgi Stop (BD, 1/1,000), PMA (Fisher, 50 ng/ml), Ionomycin (Fisher, 1µg/ml) and then fixed in IC fixation buffer (eBioscience) for 15 min at 4°C, and permeabilized for 30 min in 1X Perm/Wash buffer (eBioscience) containing indicated intracellular marker(s). For detection of transcription factors (BCL6, EOMES, FoxP3, T_bet_, TCF1), Ki67 and Granzyme B, cells were fixed and permeabilized after the surface stain with the FoxP3/Transcription Factor Staining Buffer Set following the manufacturer’s instructions (eBioscience). Stained cells were collected on BD LSR-II or Aria III and the Cytek Aurora. Data were analyzed using FlowJo version 9.6.6 or FlowJo version 10.5.3 (TriStar).

### ELISA for Parasite Antigens

Detection of antibodies against *P. yoelii* MSP1_19_ (BEI resources/ATCC, MRA-48) in mouse sera collected at indicated time points, were done by ELISA following an adapted protocol (8). Briefly, sera dilutions were incubated for 1.5 h at 37°C in high bind polystyrene microplate wells (Corning) that were first blocked with 1% BSA and then coated with MSP1_19_ (2.5 µg/ml). Total specific IgG sera antibodies were detected with horse radish peroxidase (HRP) conjugated goat anti-mouse IgG antibodies (Jackson ImmunoResearch) followed by addition of the substrate 3,3’,5,5’-tetramethylbenzidine (Sigma) and reading of absorbance at 450 nm. Endpoint titers were reported with the background absorbance of PBS coated wells subtracted.

### Statistics

Statistical significance was calculated using an unpaired Student t test with GraphPad Prism software and two-tailed P values are reported as: (*) P<0.05, (**) P<0.01, (***) P<0.001, (****) P<0.0001, (ns) P>0.1. All p values of 0.05 or less were considered significant and are referred to as such in the text.

## Results

### While Blockade of LAG-3 and PD-L1 Synergize to Enhance the Control of Blood Parasitemia, Genetic Lack of PD-1 and PD-L1 Are Compensated

**Figure 1:**
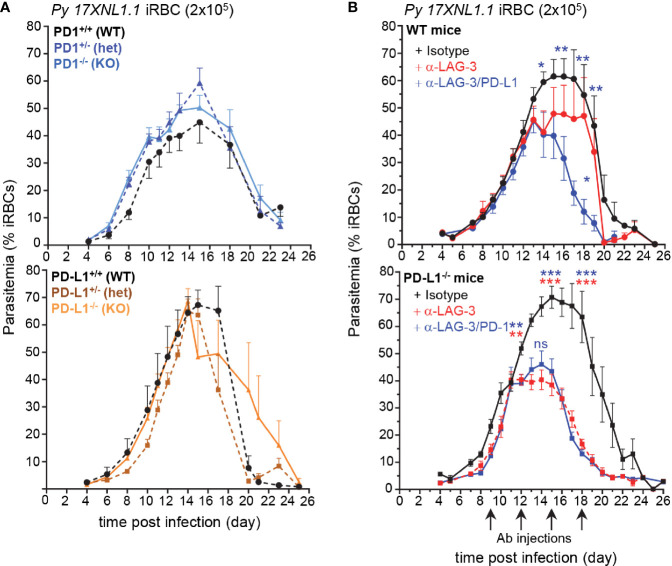
Lymphocyte Activation Gene 3 (LAG-3) and PD-L1 blockade enhances blood parasite clearance, yet PD-1-/- and PD-L1-/- mice clear parasite like WT mice. **(A)** Littermates of indicated genotypes were inoculated with 2x10^5^ *Py 17XNL1.1* iRBC i.v. Blood parasitemia was measured starting 4 days post infection and every other day until day 24–26 using YOYO-1 staining of RBC and FACS. PD1^+/+^ (n=5), PD1^+/-^ (n=5), PD1^-/-^ (n=5), PD-L1^+/+^ (n=3), PD-L1^+/-^ (n=5), PD-L1^-/-^ (n=4) **(B)** WT or PD-L1^-/-^ mice were inoculated with 2x10^5^ *Py* iRBC i.v., and 9, 12, 15, and 18 days post infection, indicated mice received 200 µg of either anti-LAG-3 (WT n=13, PD-L1^-/-^ n=12), anti-LAG-3/PD-L1(WT n= 12), anti-LAG-3/PD-1 (PD-L1^-/-^ n=10) or polyclonal rat IgG Ab (isotype, WT n=12, PD-L1^-/-^ n=12) i.v. Results show the kinetics of blood parasitemia over time determined by YOYO-1 staining of RBC collected at indicated time points. Graphs average the pool of two to three independent experiments (n=10–13) shown with SEM. P-values are indicated when applicable (*p < 0.05, **p < 0.01, ***p < 0.001) for each treatment compared to isotype (asterisk color indicate respective treatment group) or to anti-LAG-3 treatment per time point (middle blue asterisk or “ns” on graphs for anti-LAG-3/PD-L1 compared to anti-LAG3) by student’s t-test.

Overexpression of inhibitory molecules such as PD-1, LAG-3, or BTNL-2 on antigen-experienced T cells in malaria-infected patients were suggested as possible mechanisms accounting for the impaired development of effective and long-lasting adaptive immunity (7, 8, 10). Consistent with this idea, therapeutic co-blockade of PD-L1 and LAG-3 inhibitory molecules in WT mice was reported to act synergistically to decrease blood parasite growth and accelerate its clearance (8). Based on these results, we hypothesized that mice lacking the PD-1/PD-L1 inhibitory pathway should control parasite growth and clearance faster than WT counterparts. We also postulated that the therapeutic blockade of LAG-3 in PD-L1^-/-^ mice should phenocopy that of PD-L1/LAG-3 co-blockade in WT mice. To test these possibilities, we first inoculated mice genetically deficient for the genes encoding PD-1 or PD-L1 (PD1^-/-^, PD-L1^-/-^, namely KO) and their littermate controls, which possess only one functional copy of either gene (PD1^+/-^, PD-L1^+/-^, namely heterozygotes) or both copies (PD1^+/+^, PD-L1^+/+^, namely WT), with red blood cells (RBCs) infected with the non-lethal strain of *Plasmodium, P. yoelii 17XNL (Py)* (**Figure 1A**). Contrary to expectations, the kinetics of *Py* growth and elimination was similar between all these groups, suggesting the existence of compensatory mechanisms making PD1^-/-^ and PD-L1^-/-^ mice as resistant to *Py* infection as WT mice. We next injected WT and PD-L1^-/-^ mice with anti-LAG-3 and anti-LAG-3/PD-L1 or anti-LAG-3/PD-1 mAbs, respectively (**Figure 1B**). LAG-3 blockade in PD-L1^-/-^ mice promoted reduced parasitemia to an extent that was comparable to that of WT mice treated with anti-PD-L1/LAG-3 mAbs. This result was also confirmed in female PD-L1^-/-^ mice (**Supplementary Figure 1A**). LAG-3 blockade in WT mice promoted only a partial reduction of parasitemia compared to co-blockade with anti-PD-L1 mAb. Further, treatment of PD-1^-/-^ mice with anti-LAG-3 mAb confirmed that LAG-3/PD-L1 co-blockade in WT mice resulted in a synergistic enhancement of parasite elimination (**Supplementary Figure 1B**). Therapeutic blockade of PD-1 in PD-L1^-/-^ mice, which neutralizes PD-1/PD-L2 interactions, resulted in delayed blood parasite clearance, consistent with a prior report (14) showing that blockade of the PD-1/PD-L2 axis prevents effective parasite control (**Supplementary Figure 1C**). Interestingly, blood parasitemia was comparable in anti-LAG-3/PD-1 versus anti-LAG-3 treated PD-L1^-/-^ mice, suggesting that inhibition of LAG-3 is dominant over that of PD-1/PD-L2. Collectively, these findings i) confirm the importance of PD-1-dependent and LAG-3 inhibitory pathways in resistance to malaria infection and ii) reveal that blockade of a specific inhibitory pathway, here LAG-3, can have distinct impacts depending on genetic or functional deficiencies in other related pathways, here PD-L1 or PD-L2.

### B and CD4+ T Cell Responses in PD1-/-, PD-L1-/- and Heterozygous Littermates Are Comparable to That of Wild Type Mice

Since PD1^-/-^, PD-L1^-/-^ and WT mice exhibited similar resistance to *Py* infection as WT mice, we next wondered whether adaptive cellular immune responses may still differ between these mice. As an initial step, we characterized the immune response in *Py*-infected WT mice over the ~25 day infection course. Since B cells play an essential role in protective immunity against malaria (8, 15), we carried out an in-depth kinetic analysis of this cellular compartment in the spleen of *Py*-infected mice at 7.5, 12.5, and 15.5 days post infection (**Supplementary Figure 2**). Using advanced flow-cytometry gating strategies to define subsets of splenic B cells (16), we subdivided the CD19^+^ B cells based on IgD and IgM cell-surface expression (**Supplementary Figure 2A**). By day 7.5, and peaking at day 12.5 of *Py*-infection, ~40% of CD19^+^ B cells underwent isotype switching, becoming IgM^low^IgD^low^, while proportions of all other B cell subpopulations decreased. IgM^low^IgD^hi^ B cells modestly diminished over the infection course, and represented ~50% of CD19^+^ B cells that include type I follicular B cells (FOL I) (**Supplementary Figure 2B**). We also noted reduction of IgM^hi^IgD^low^ B cells, which likely converted to marginal zone (MZ) B cells and B1 B cells. Proportions of all B cell transitional stages (T1, T2, T3) decreased during infection, yet marginal zone precursors (MZPs) and type II follicular B cells (FOL II) were largely maintained. The most noticeable changes occurred in the germinal centers (GC, GL7^+^CD95^+^) representing ~15%–23% of splenic B cells between day 7.5 and 15.5 post infection (**Supplementary Figure 2C**). Plasmablast generation (CD138^+^PNA^+^) quickly increased following *Py*-infection, peaking by day 7.5, and represented ~7-10% of the splenic B cells. Production of effective GC and plasma B cell responses requires CD4^+^ T_FH_ cell responses (17). As expected, a substantial proportion of T_FH_ cells (ICOS^+^Bcl6^+^) formed by 7.5 days post infection (~30% of CD4^+^ T cells) while only few follicular regulatory T cells (T_FR,_ Foxp3^+^Bcl6^+^) were generated by this time point, representing ~7% of Foxp3^+^ T cells and ~0.5-1% of all CD4^+^ T cells (**Supplementary Figure 3A**).

**Figure 2:**
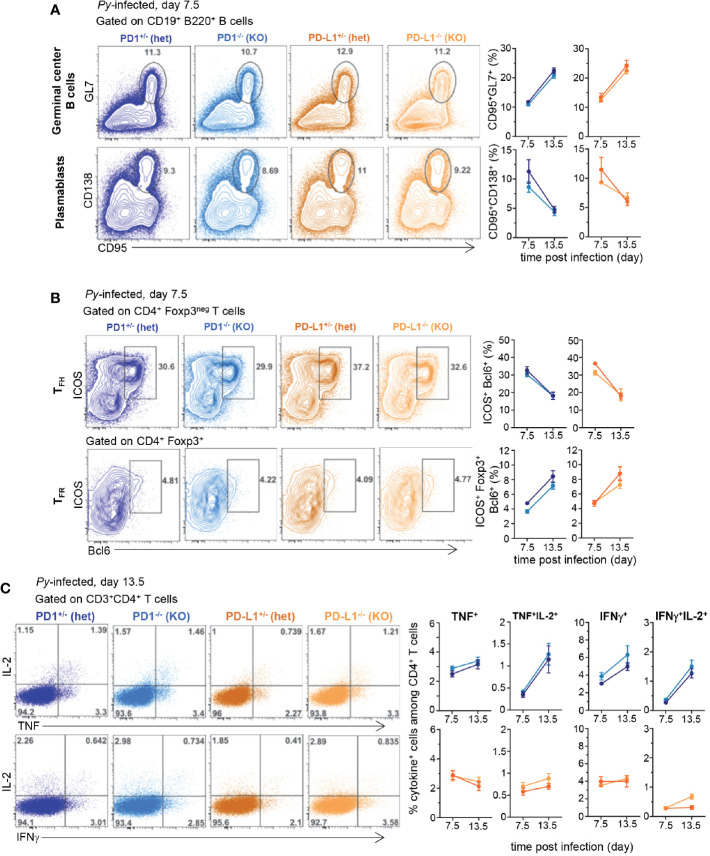
B and CD4^+^ T cell responses are comparable in *Plasmodium yoelii*-infected PD1^-/-^, PD-L1^-/-^, and heterozygous counterparts littermates. Littermates of indicated genotypes were inoculated with 2x10^5^ *Py 17XNL1.1* (iRBC) i.v. **(A)** Spleens from *Py*-infected mice were harvested 7.5 and 13.5 days post infection and stained for CD19, B220, GL7, CD138, CD95 to monitor B cell responses and **(B)** spleen cells were stained for CD4, CD3, Foxp3, Bcl6, ICOS to monitor CD4^+^ T_FH_ and T_FR_ cellular responses by flow cytometry. **(C)** Spleen cells from either uninfected, day 7.5 or 13.5 *Py*-infected (2x10^5^ iRBC, i.v.) PD-1^-/-^ (KO), PD-1^+/-^ (hets), PD-L1^-/-^ (KO), PD-L1^+/-^ (hets) mice were incubated with PMA/ionomycin for 4 h and then stained for cell-surface CD3, CD4 and CD8 and intracellular cytokines IL-2, IFNγ, and TNF. Frequencies of cytokine-producing cells among indicated T cell subset are shown. In all experiments, representative FACS dot plots of two independent replicate experiments are presented (n=3–12). Graphs show average results from experiments with SEM. Student t-test was conducted between groups and p-values are indicated when applicable.

Using the knowledge gained from this comprehensive analysis of the robust B cell and associated CD4^+^ T_FH_ cell responses induced after blood stage *Py* infection, we next compared these responses at key time points (days 7.5 and 13.5) in *Py*-infected PD1^-/-^, PD-L1^-/-^ and WT mice (**Figure 2**). GC formation and plasmablast, CD4^+^ T_FH_ and T_FR_ cell differentiation at 7.5 and 13.5 days post infection were equivalent between all groups of mice (**Figures 2A, B** and **Supplementary Figure 3B**). We also assessed if T cells isolated from the various groups of *Py*-infected mice were functionally distinct compared to non-infected counterparts, by measuring the production of effector cytokines (IL-2, TNF, IFNγ). In splenocytes from naïve or *Py*-infected WT mice, the proportion of single and double cytokine-secreting CD4^+^ and CD8^+^ T cells after short *ex vivo* restimulation with PMA/ionomycin, was significantly reduced compared to that of uninfected controls (by ~75%–90%) (**Supplementary Figure 3C**). In PD-1^-/-^, PD-L1^-/-^ and heterozygous mice, we measured a comparable loss of cytokine-secreting capacity compared to that of naïve mice, yet comparable proportions were found in all groups (**Figure 2C** and **Supplementary Figure 3D**). Thus, altogether, the lack of observed differences in i) blood parasite growth and elimination, ii) B and CD4^+^ T_FH_ cell responses and iii) production of effector cytokines across the distinct experimental groups, suggest a relatively modest contribution of the PD1/PD-L1 pathway in natural protection against blood stage malaria. These results are in contrast with prior published reports using PD-L1 Ab blockade in WT mice (8, 13).

### LAG-3 Neutralization in PD-L1-/- Mice Neither Promotes Greater CD4+ TFH, GC and Plasmablast Cellular Responses nor Higher Parasite-Specific Antibody Titers With Enhanced Protective Capacity

**Figure 3:**
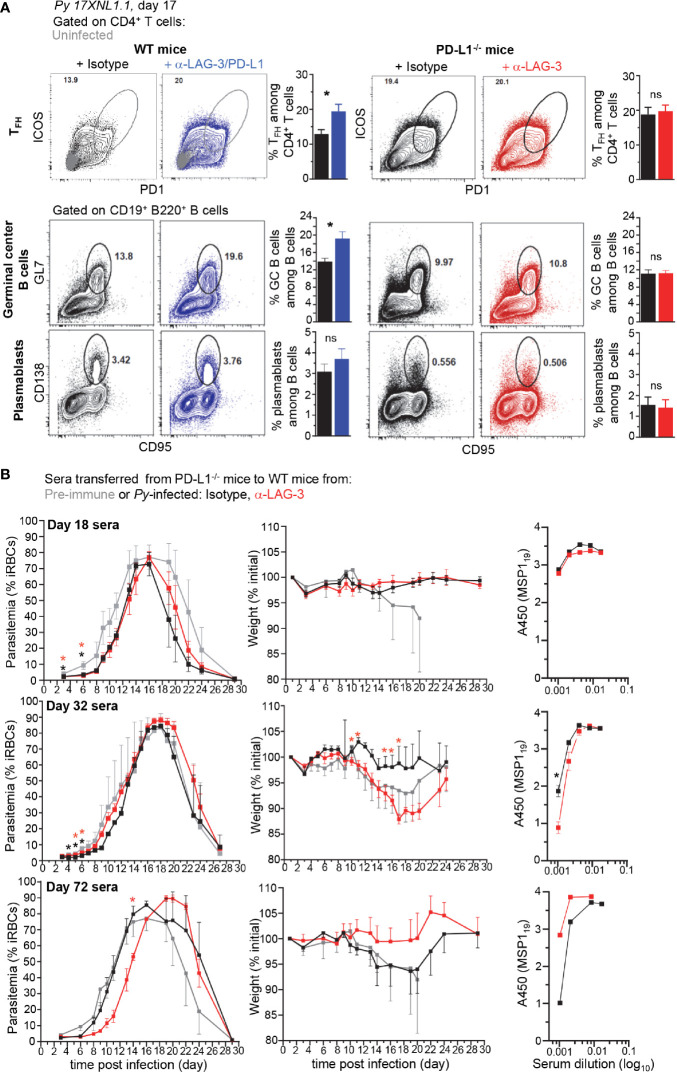
Lymphocyte Activation Gene 3 (LAG-3)/PD-L1 therapeutic blockade enhances T_FH_ and germinal center (GC) responses while LAG-3 blockade in PD-L1^-/-^ mice does not. **(A)** Wild-type (WT) B6 or PD-L1^-/-^ mice were inoculated with 2x10^5^ *Py 17XNL1.1* iRBC i.v. and 9, 12, 15, and 18 days post infection, indicated mice either received 200 µg of either anti-LAG-3/PD-L1, anti-LAG-3 or isotype Ab i.v. Spleens were harvested 17 days post infection and stained for CD4, CD3, ICOS, PD-1 to monitor CD4^+^ T_FH_ cell response (ICOS^+^PD-1^hi^ corresponds to ICOS^+^Bcl6^hi^ cells) (top panel) and for CD19, B220, GL7, CD138, CD95 to monitor B cell responses (lower panels). Representative FACS dot plots of one to two independent replicate experiments are presented (n=4–10), with an overlay of representative uninfected mouse in the WT top panel. Graphs average each experiment shown with SEM. Student t-test was conducted between groups and p-values are indicated when applicable (*p < 0.05). **(B)** 150 µl of sera harvested from mice 18, 32, or 72 days post *Py*-infection was transferred to naïve WT mice (3-5 mice/group) that were subsequently infected with 2x10^5^ *Py 17XNL1.1* iRBC i.v. Blood parasitemia and weight was monitored over 28 days. Titers of *Py* MSP1_19_-specific IgG antibodies in indicated sera was measured by ELISA and reported as background subtracted A_450_ values. p-values are indicated when possible compared to pre-immune treatment or to isotype treatment by student t-test. Asterisk color indicate respective treatment group (*p < 0.05).

Since the reported mechanism by which LAG-3/PD-L1 blockade in WT mice enhances parasite clearance is through greater GC B and CD4^+^ T_FH_ cell responses and higher titers of parasite-specific Abs (8), we hypothesized that LAG-3 neutralization in PD-L1^-/-^ mice may work through a similar mechanism. We monitored both CD4^+^ T_FH_ and B cell GC and plasmablast responses in PD-L1^-/-^ and WT mice treated with anti-LAG-3 or anti-LAG-3/PD-L1, respectively (**Figure 3A**). While as expected, blockade of LAG-3 and PD-L1 in WT mice promoted significantly enhanced T_FH_ and GC B cell responses, we did not find any differences in these cell populations in anti-LAG-3-treated PD-L1^-/-^ mice. Of note, spleen weight, and total or hematopoietic-derived (CD45^+^) cell counts per spleen were comparable in WT and PD-L1^-/-^ mice undergoing the various treatments, likely ruling out that the lack of differences in cell subset proportions in the distinct experimental groups is due to differences in absolute cell numbers (**Supplementary Figure 4**).

We next conducted experiments to assess if despite the lack of obvious differences in B and CD4^+^ T_FH_ cell responses in PD-L1^-/-^ mice, parasite-specific Abs produced in anti-LAG-3-treated mice may still confer better protection and exhibit higher serum titers compared to isotype-treated counterparts (**Figure 3B**). We transferred sera (150 µl/mouse, one time) isolated from anti-LAG-3 or isotype Ab-treated PD-L1^-/-^ mice 18, 32, or 72 days post *Py*-infection, into WT B6 recipient mice that were subsequently infected with *Py*. Data reveal a delayed onset and peak blood parasitemia, and lower weight loss in mice treated with day 72 sera, but no beneficial trends were observed in day 18 or 32 sera transfers. This result was extended in sera isolated from day 32 *Py*-infected WT mice treated with anti-LAG-3/PD-L1 or isotype control Abs, suggesting that, at least under these experimental conditions, transfer of parasite-specific Abs from *Py*-infected mice failed to confer significantly distinct levels of protection to recipient mice (**Supplementary Figure 5A**). Yet, immune sera from day 72-infected WT mice did confer increased protection and prevented weight loss compared to pre-immune sera, validating our serum transfer experiments and read-outs. Along these lines, only sera from day 72 anti-LAG-3, but not isotype-treated infected PD-L1^-/-^ mice, delayed parasitemia in recipient mice that were subsequently infected, suggesting that PD-L1^-/-^ mice are unable to develop long-term parasite-specific protective Abs (**Figure 3B**). Comparative day 32 sera transfers between isotype Ab-treated WT and PD-L1^-/-^ mice showed no differences in parasitemia in recipient mice, yet slightly better *Py* clearance and lower weight loss for day 72 sera from WT mice was observed compared to that of PD-L1^-/-^ mice (**Supplementary Figure 5B**). This result was consistent with the idea that PD-L1^-/-^ mice do not develop effective parasite-specific protective Abs.

To provide additional evidence to these findings, we also measured parasite-specific Ab titers using the recombinant merozoite surface antigen MSP1_19_, a major blood stage parasite antigen (Ag) (**Figure 3B** and **Supplementary Figure 5A**). Consistent with the serum transfer experiments, titers of parasite-specific Abs specific for MSP1_19_ in the sera of anti-LAG-3 versus isotype Ab-treated PD-L1^-/-^ mice (day 18, 32) did not reveal significant differences. Slightly higher Ab titers were nevertheless measured in day 72 sera from anti-LAG-3-treated PD-L1^-/-^ mice, in line with the observed delayed parasitemia results (**Figure 3B**). Likewise, slightly higher parasite-specific Ab titers were quantified in day 72 sera from anti-LAG-3/PD-L1-treated WT mice (**Supplementary Figure 5A**).

These results suggest that the mechanism, by which LAG-3 blockade in PD-L1^-/-^ mice and LAG-3/PD-L1 co-blockade in WT mice enable the rapid control of blood parasitemia, is unlikely to involve the production of higher titers of parasite-specific Abs. Also consistent with prior studies, our data show that peak production of such protective Abs most likely occurs within 2-3 months after infection, but not at early stages (18).

### CD4+ T Cells From Anti-LAG-3-Treated PD-L1-/- Mice Express More Effector Cytokines Compared to Untreated Counterparts

**Figure 4:**
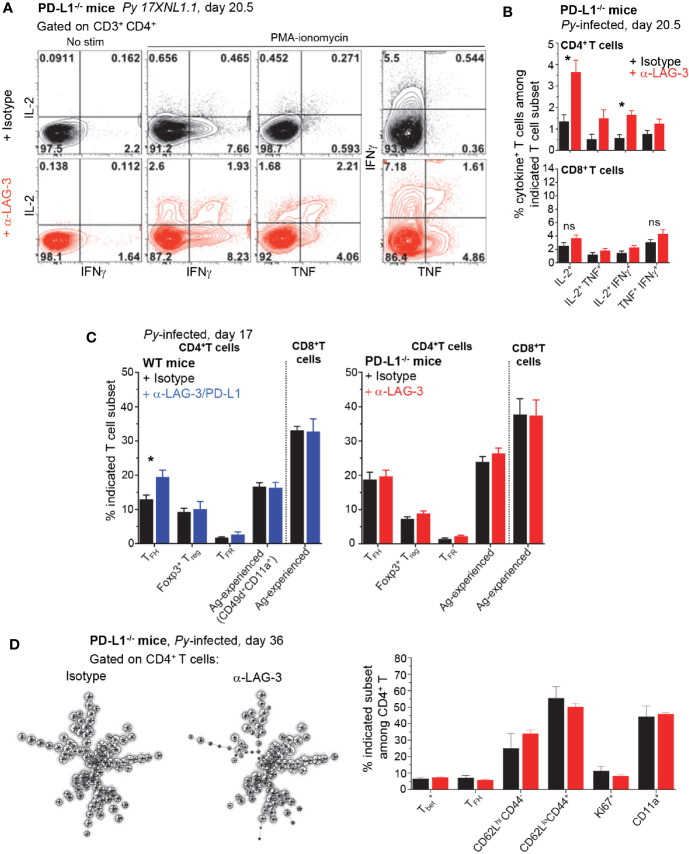
Lymphocyte Activation Gene 3 (LAG-3) blockade in PD-L1^-/-^ mice partially restores CD4^+^ T cell dysfunction. Spleen cells from day 20.5 *Py*-infected PD-L1^-/-^ or WT mice, treated with either anti-LAG-3/PD-L1, anti-LAG-3 or isotype Ab i.v. (as indicated, on days 9, 12, 15) were stained for cell-surface CD3, CD4 and CD8, Foxp3, Bcl6, ICOS and intracellular cytokines IL-2, IFNγ, and TNF after incubation with PMA/ionomycin for 4 h (T cell mix). **(A)** Representative FACS dot plots of two independent experiments are shown with the frequencies of cytokine-producing cells among CD4^+^ T cells (n=3–5). **(B)** Graphs of the average frequencies of cytokine-producing cells among CD4^+^ T cells (shown in **(A)**) and CD8^+^ T cells with SEM. P-values are indicated when applicable. **(C)** Distribution of known splenic T cell subsets as indicated on day 17 post infection, T_FH_ data are from the same experiment shown in Figure 7. Graphs average one to two independent experiments (n=4-5) shown with SEM. **(D)** Spleen cells from day 36 PD-L1^-/-^ *Py*-infected mice treated with either anti-LAG-3 or isotype Ab i.v. (on days 9, 12, 15) were stained for lineage markers (CD8, CD4, CD3, Bcl6, T_bet_) and functional markers (CD62L, CD44, CD11a) with a 24 color panel depicted in S3A Fig. FlowSOM analysis (left) on pooled CD4^+^ T cells (n=5) and summary bar graphs from each treatment group are shown (right). Distribution of known splenic T cell subsets as indicated on day 36 post infection. Student t-test was conducted between groups and p-values are indicated when applicable (*p < 0.05).

Our results so far led us to hypothesize that blockade/disruption of the LAG-3 and PD-1/PD-L1 pathways enhances rapid parasite clearance *via* CD4^+^ T cells, independent from humoral response. Since therapeutic blockade of these pathways was also shown to restore malaria-induced T cell dysfunction (8, 9), we next tested if T cells isolated from LAG-3-treated PD-L1^-/-^ mice were more responsive to broad PMA/ionomycin stimulation than control groups at day 17, when rapid parasite elimination is observed (**Figure 1**). We report significant differences by a factor of 2-3 fold, in the relative proportion and numbers of single (IL-2^+^) and double (IL-2/TNF^+^, IL-2/IFNγ^+^, IFNγ/TNF^+^) cytokine-secreting CD4^+^, but not CD8^+^, T cells in the anti-LAG-3-treated group compared to the control group, consistent with our hypothesis (**Figures 4A, B**). While a higher proportion of anti-LAG-3-treated CD4^+^ T cells accumulated effector and proliferative cytokines, the distribution of the various phenotypic and functional subsets of CD4^+^ and CD8^+^ T cells (T_FH_, T_reg_, T_FR_, Ag-experienced) remained similar between all experimental groups and conditions (**Figures 4C** and **3A**). High dimensional flow-cytometry analysis of the CD4^+^ T cells at later stages (day 36) with 25 colors (**Supplementary Figure 6A**), also failed to further reveal any differences in subsets of CD4^+^ T cells (T-bet, T_FH_), activation/memory status (CD62L, CD44, CD11a) and proliferation (Ki67) between anti-LAG-3 and control isotype Ab-treated PD-L1^-/-^mice (**Figure 4D** and **Supplementary Figure 6B**). In summary, LAG-3 blockade contributes to rescuing malaria-induced CD4^+^ T cell dysfunction in PD-L1^-/-^ mice, but does not appear to modulate their ability to differentiate into various functional and memory subsets of T cells.

### Antigen-Experienced $T_{\text{bet}}^{+}$ CD8^+^ and $T_{\text{bet}}^{+}$, T_FH_ and T_FR_ CD4^+^ T Cells Are the Major LAG-3-Expressing Cell Subsets

**Figure 5:**
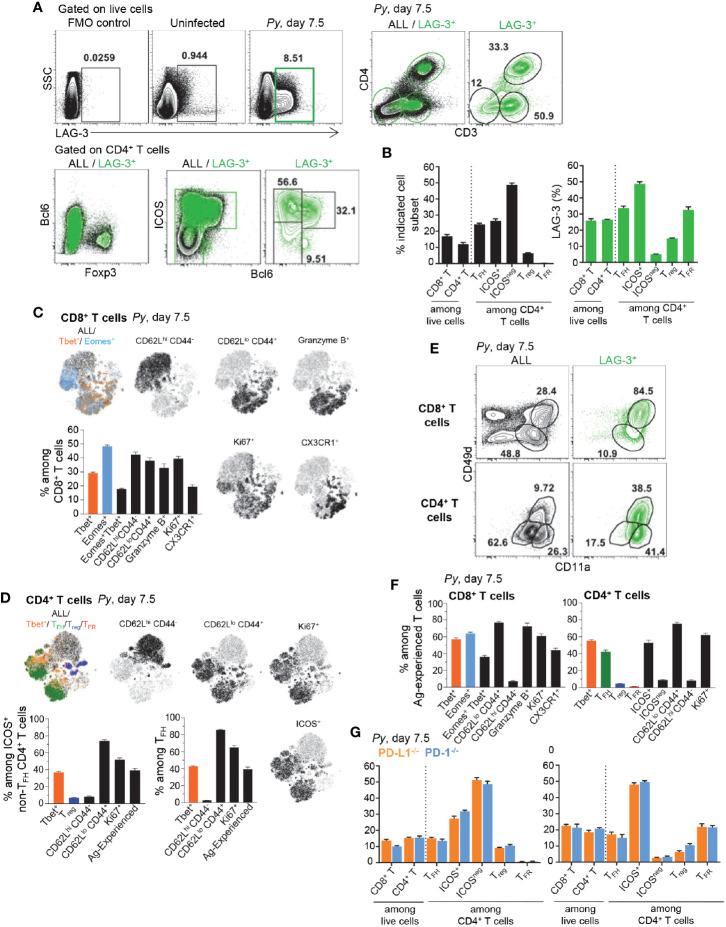
Lymphocyte Activation Gene 3 (LAG-3) is expressed on activated CD4^+^ and CD8^+^ T cells during *Py* infection. Spleen cells from day 7.5 *Py*-infected mice were stained for lineage markers (CD8, CD4, CD3, Foxp3, Bcl6) and activation/inhibitory markers (LAG-3, CD11a, CD49d), or with a 26 color panel in S3A Figure **(C, D, F)**. **(A, B)** FACS dot plots of the phenotype and **(B)** proportion of LAG-3-expressing cells among the indicated T cell subsets in WT mice. **(C, D)** t-SNE overlay representation of indicated CD8^+^ **(C)** and CD4^+^ **(D)** T cell subsets from a pool of five infected WT mice with bar graphs summarizing the proportions of indicated subsets with SEM. **(E)** Proportion of Ag-experienced (CD11a^+^CD49d^+^) CD8^+^ and CD4^+^ T cells on all or LAG-3^+^ cells. **(F)** Proportions of indicated subsets among Ag-experienced CD8^+^ and CD4^+^ T cells from the pool of five infected WT mice with SEM. **(G)** proportion of LAG-3-expressing cells among the indicated T cell subsets in PD-L1^-/-^ or PD-1^-/-^ mice. Overall, representative FACS dot plots of two independent replicate experiments are presented for one of three to five mice. Graphs show average results from experiments with SEM. P-values are indicated when applicable.

To gain further insight into how LAG-3 blockade contributed to reduced blood parasitemia, we next investigated which cells express the LAG-3 receptor during *Py* infection (**Figure 5**). LAG-3-expressing spleen cells represented ~8.5% of live cells by day 7.5 post-*Py* infection and largely consisted of T cells (>80%) that make up ~26% of splenic cells, distributed among CD4^+^ and CD8^+^ T cells (**Figures 5A, B**). Among LAG-3^+^ CD4^+^ T cells, a majority were activated ICOS^+^ conventional T cells (~48%), T_FH_ cells (~33%) and T_FR_ cells (~32%). Some Foxp3^+^ T_reg_ cells also expressed LAG-3 but only represented a small proportion of all CD4^+^ T cells (<6%). Using high dimensional flow cytometry we assessed cell-surface lineage, activation and intracellular functional markers, as well as lineage specifying transcription factors. We found that activated and proliferating (CD62L^lo^CD44^hi^Ki67^+^) CD8^+^ T cells distributed into $T_{\text{bet}}^{+}$, Eomes^+^ and $T_{\text{bet}}^{+}/\text{Eome}s^{+}$ effector cells (**Figure 5C**). $T_{\text{bet}}^{+}$ cells expressed high levels of the cytolytic marker granzyme B and cell-surface marker CX3CR1, which are features of terminally differentiated effector cells (19). Interestingly, activated and proliferating CD4^+^ T cells mostly included $T_{\text{bet}}^{+}$ and T_FH_ cells, both of which expressed high levels of the cell-surface marker ICOS (**Figure 5D**). In contrast, ICOS^neg^ CD4^+^ T cells were not activated (CD62L^hi^CD44^lo^Ki67^neg^) (**Supplementary Figure 6C**). Interestingly, using CD49d and CD11a cell-surface markers as a proxy for activated parasite-specific T cells, we found a substantial proportion of LAG-3^+^ T cells expressed both markers (~84% and ~38% respectively, for CD8^+^ and CD4^+^ T cells) and >90% also upregulated the adhesin CD11a (**Figure 5E**). A large proportion of CD49d^+^/CD11a^+^ CD8^+^ and CD4^+^ T cells were activated and proliferated (CD62L^lo^CD44^hi^Ki67^+^), and expressed T_bet_, Eomes and were T_FH_ (for CD4^+^ T cells) (**Figure 5F**). Lastly, consistent with our prior results (**Figure 2**), analysis of LAG-3^+^ T cells in *Py*-infected (day 7.5) PD-L1^-/-^ and PD-1^-/-^ mice failed to reveal any differences of subset distribution between them, and compared to WT counterparts (**Figures 5G, B**). Thus, these data support a model in which the largest proportion of LAG-3^+^ cells are T cells that are likely parasite-specific, and include effector $T_{\text{bet}}^{+}$ CD8^+^ T cells and $T_{\text{bet}}^{+}$, T_FH_ and T_FR_ CD4^+^ T cells.

The major ligand for LAG-3 is MHC-II. In an effort to provide additional insights into which cells may be regulating LAG-3^+^ CD4^+^ T cells, we analyzed MHC-II^+^ cell subsets in *Py*-infected mice (**Supplementary Figure 6D**). While the vast majority of MHC-II^+^ cells were B cells (50%), we observed that DCs (1.7%), Ly6C^+^ monocytes (2%) and neutrophils (2.7%) also expressed MHC-II. Expression levels were highest on DCs, followed by B cells, in both uninfected and infected mice, and *Py*-infection induced upregulation on most of these cell subsets. Comparison of MHC-II expression on these cells in *Py*-infected PD-L1^-/-^, PD-1^-/-^ and WT mice highlighted slight but significantly increased frequencies in MHC-II^+^ DCs and monocytes in PD-L1^-/-^and PD-1^-/-^ compared to WT mice (**Supplementary Figure 6E**). However, the relative distribution of MHC-II^+^ cells was the same in all groups. Thus altogether, these results suggest that B cells and DCs may represent the most likely regulators of activated LAG-3^+^ CD4^+^ T cells.

### Anti-LAG-3-Mediated Parasite Elimination Is Independent From CD8+ T Cells

**Figure 6:**
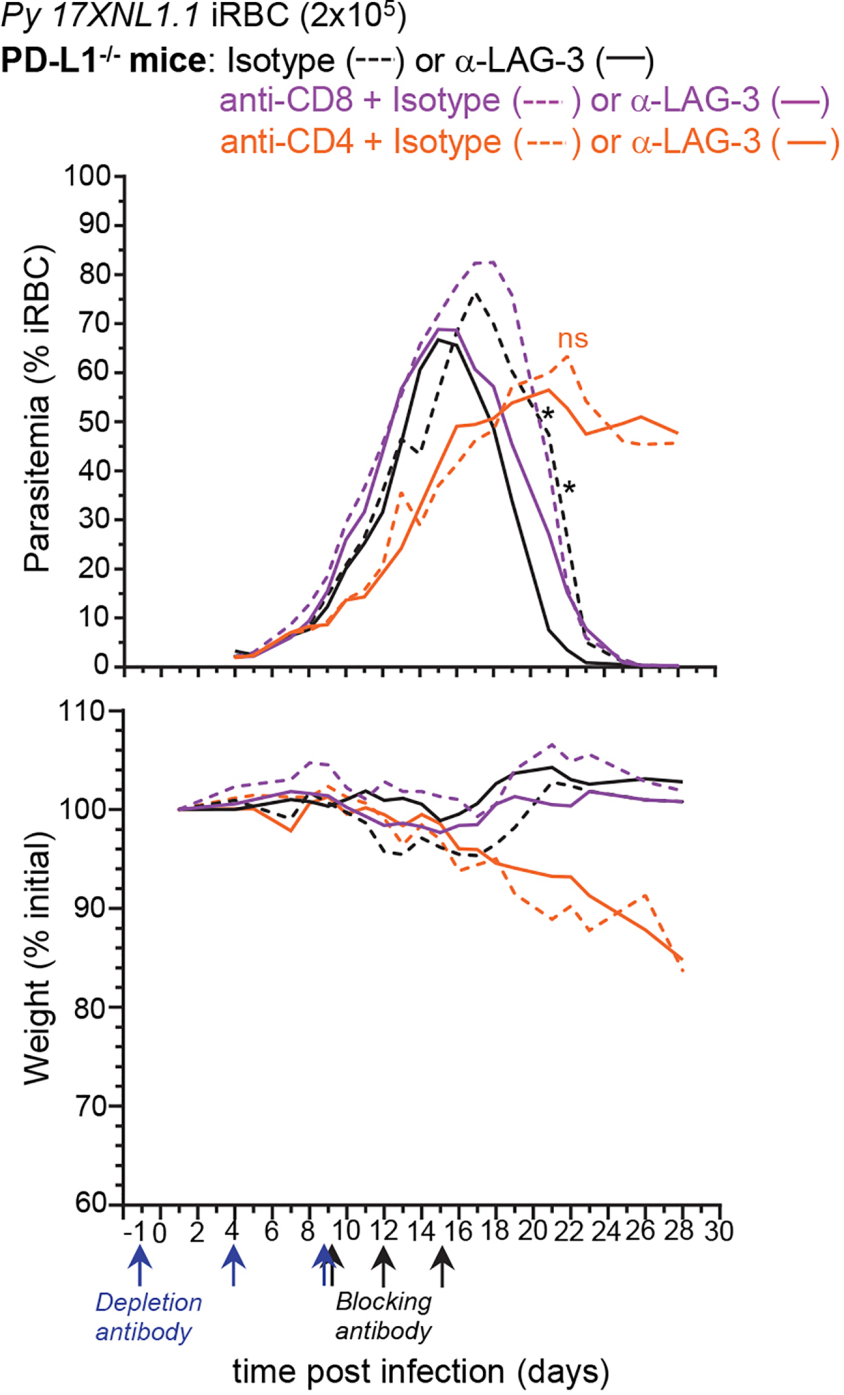
Lymphocyte Activation Gene 3 (LAG-3)-mediated enhancement of anti-parasite immunity is not dependent on CD8^+^ T cells. PD-L1^-/-^ mice were injected i.p. with PBS or 150µg of either anti-CD4 or anti-CD8β depleting mAbs as depicted and inoculated with 2x10^5^ *Py 17XNL1.1* iRBC i.v. Mice also received either 300 µg anti-LAG-3 or isotype Ab i.v. on day 9, 12, and 15 post *Py* infection. Results show blood parasitemia measured by YOYO-1 staining of RBC, and change in initial weight (lower panel) over time starting on day 4 post *Py* infection. Anti-CD8β + isotype (n=3), anti-CD4 + isotype (n=7), Anti-CD8β + anti-LAG3 (n=5), anti-CD4 + anti-LAG3 (n=8), isotype (n=4), anti-LAG3 (n=5). Graphs are representative of two independent experiments (n=3–4) shown with SEM. Student t-test was conducted between each treatment compared to isotype (asterisk color indicate respective group), p-values are indicated when applicable (*p < 0.05).

Since the major LAG-3^+^ cell types are CD8^+^ and CD4^+^ T lymphocytes, we next tested if selective depletion of either T cell subset would abrogate the LAG-3-mediated protective effect (**Figure 6**). PD-L1^-/-^ mice were either injected with anti-CD8β or anti-CD4 depleting mAbs or PBS prior to infection with *Py*, and 9, 12, and 15 days post-infection, mice received anti-LAG-3 or control isotype Abs. We monitored weight and parasitemia every other day until the undepleted isotype Ab-treated control groups were negative for blood parasitemia. LAG-3 blockade in CD8^+^ T cell-depleted PD-L1^-/-^ mice enhanced the control and clearance of blood parasitemia similarly to that of undepleted control groups, indicating that anti-LAG-3 blockade does not act on CD8^+^ T cells. In contrast, CD4^+^ T cell-depleted mice failed to control parasite growth and developed chronic parasitemia, losing >10% of initial body weight 20 days post-infection whether treated with anti-LAG-3 or control isotype Abs, and consistent with their requirement for *Py* parasite clearance (8). Taken together with the finding that LAG-3 is mostly expressed on T cells (**Figure 5**), these results suggest that the anti-LAG-3-mediated therapeutic effect on blood parasite elimination is likely to occur by blocking LAG-3 on parasite-specific $T_{\text{bet}}^{+}$ and/or T_FH_ CD4^+^ T cells, but not on CD8^+^ T cells.

## Discussion

In this work, we investigated the mechanisms by which blockade of the inhibitory receptors LAG-3 and PD-L1 promote more effective control and clearance of non-lethal blood stage murine *P. yoelii* malaria. We reveal that LAG-3 neutralization in mice that are genetically deficient for PD-1/PD-L1 did not promote stronger CD4^+^ T_FH_ and GC B cell responses as it was reported in anti-PD-L1/LAG-3 treated WT mice (8). Consistent with this result, convalescent sera (day 32) transfers from anti-LAG-3 treated PD-L1^-/-^ mice and anti-PD-L1/LAG-3 treated WT compared to control isotype Ab treated mice failed to confer distinct levels of protection against *Py* infection in naïve recipient mice. Additionally, sera titers of parasite (MSP1_19_)-specific Abs were comparable in all immunized groups of mice. We further found that LAG-3, at the time of Ab blockade, is mostly expressed by T cells, with a large majority consisting of activated, proliferating Ag-experienced (CD49d^+^CD11a^+^) T cells that include $T_{\text{bet}}^{+}\;CD8^{+}$ and ICOS^+^, T_FH_ and T_FR_ CD4^+^ T cells. We show that while both subsets of T cells express LAG-3, the protective effect of LAG-3-blockade does not require CD8^+^ T cells and thus is most likely mediated through CD4^+^ T cells. We also report that LAG-3 blockade enhances CD4^+^ T cell effector functions in infected mice. Thus, we propose a model in which inhibition of LAG-3 on parasite-specific CD4^+^ T cells unleashes their ability to produce effector cytokines, altogether contributing to achieve superior levels of protection in malaria-infected hosts, independent from parasite-specific humoral responses.

Our finding that mice with genetic deficiency in the PD-1/PD-L1 pathway are as susceptible to *Py* malaria infection as their WT counterparts highlights some unexpected discrepancies with prior work showing that therapeutic mAb blockade of PD-L1 in WT mice promotes enhanced control and clearance of blood parasitemia (8, 13). The fact that i) the kinetics of infection was comparable in *Py*-infected PD-1^-/-^, PD-L1^-/-^, heterozygous and WT littermate mice, and ii) we did not find any differences in either T, B cell or Ab responses despite extensive analyses, suggests that genetic deficiency in PD-1 and PD-L1 may favor the utilization of distinct mechanisms of resistance against this infection through otherwise redundant pathways. The indistinguishable kinetics of parasitemia in PD-1^-/-^ and PD-L1^-/-^ mice together with the LAG-3 blockade experiment support such an interpretation, since LAG-3 neutralization in PD-L1^-/-^ mice promotes remarkable growth control and clearance of blood parasites, which in WT mice is only achieved upon co-blockade with PD-L1. A similar observation has been reported in a model of ovarian cancer in which therapeutic co-blockade of PD-1/LAG-3/CTLA-4 in WT mice leads to two times fewer tumor free mice compared to anti-LAG-3/CTLA-4 treatment in PD-1^-/-^ mice (20). These results further illustrate a more generally accepted dogma that mice genetically deficient in important molecules often use compensatory defense mechanisms that rely on other redundant pathways. Thus, and more generally, it is conceivable that any genetic deficiency for specific inhibitory pathways is likely to alter the outcomes of specific therapies targeting these important pathways in immune defense and homeostasis.

While the genetic deficiency in PD-L1 versus therapeutic mAb blockade of PD-L1 in WT mice clearly results in different outcomes for *Py* parasitemia and immune responses, our experiments using anti-LAG-3 mAb blockade raises an important point related to the underlying mechanisms of increased resistance to *Py* infection. The Butler study reported significantly stronger T_FH_, GC and plasmablast responses, as well as higher titers of MSP1_19_ parasite-specific Abs in anti-PD-L1/LAG-3-treated WT mice compared to control groups. Whereas we did confirm enhanced T_FH_ cell and GC responses in anti-PD-L1/LAG-3 treated WT mice, this resulted neither into higher parasite (MSP1_19_)-specific Ab titers, nor into a better ability to confer protection to sera-transferred recipient mice infected with *Py*. We extended these findings to anti-LAG-3 versus control isotype Ab treated PD-L1^-/-^ mice, suggesting that parasite-specific Ab responses are unlikely to account for the enhanced anti-parasitic response that follows LAG-3 mAb blockade. While our serum transfer experiments were conducted according to the Butler study, i.e., single transfer of 150 µl of pooled sera from convalescent mice (day 32) prior to *Py* infection of recipient mice, most previous studies showing significant protection against malaria upon serum transfer, used 200 µl and at least three serial transfers at the beginning of infection (21, 22). We also transferred sera isolated from mice at different time points post *Py* infection (day 18, 32 and 72) following the rationale that, if Abs are indeed mediating an effective anti-parasite response, this effect should be even clearer at early time points of infection when the differences between anti-LAG-3 and control isotype Ab treated mice are the greatest (day 18). Yet our results did not support such an interpretation. While we observe that sera from day 72 convalescent mice -a time point at which peak parasite specific Abs are produced (18)- conferred better protection to recipient mice compared to pre-immune sera, we did not find i) any significant differences in the ability to confer parasite-specific protection or ii) higher parasite-specific Ab titers in anti-LAG-3 or anti-PD-L1/LAG-3 treated PD-L1^-/-^ or WT mice, respectively. Since we did not observe differences, we did not extend our sera analysis to potential differences in isotype switching among the *Py-*specific IgGs. Yet it is worth noting that previous work did report that *Py*-specific IgG2a Abs conferred or were associated with better protection, compared to other isotypes, presumably through the recognition of a similar set of *Py* antigens (23–25). It is therefore conceivable that PD-L1 and/or LAG-3 blockade does lead to the production of a distinct set of IgG isotypes despite the lack of functional differences. Interestingly, in sera from *Plasmodium falciparum* (*Pf*)-infected children, higher IgG3 and to some extent IgG1 and IgG4 levels were associated with protective *Pf* antigens boosted upon RTS/S vaccination, while IgG2 correlated with increased malaria risk (26). *Pf* (AMA-1)-specific IgG1 and IgG3 Ab titers are also associated with immunity above a certain age (27), consistent with the notion that IgG isotypes, in addition to Ag specificity, are likely associated with parasite-specific humoral immunity.

We also note that our anti-LAG-3 treatment regimen was only 4 consecutive injections, compared to 7 in the Butler study. While it is possible that this accounts for some differences in the outcomes, the day 32 sera transfers conducted in this prior work seem unlikely to reflect fully the protective mechanisms at work during anti-LAG-3 treatment between day 9 and 18 post *Py* infection. Taken together, we favor the interpretation that LAG-3 neutralization leads to improved control of parasitemia by rescuing functional effector CD4^+^ T cell responses, independent of parasite-specific Ab responses. A high proportion of activated proliferating (CD62L^lo^CD44^hi^Ki67^+^) Ag-specific (CD49d/CD11a^+^) effector $\left(T_{\text{bet}}^{\text{hi}}\right)\;\text{CD}4^{+}$ T cells are observed among LAG-3^+^ CD4^+^ T cells. We found that a greater frequency of these cells secrete effector cytokines (IFNγ, TNFα, IL-2) in anti-LAG-3-treated versus control mice. These effector cytokine-secreting CD4^+^ T cells may also promote the influx and activation of higher numbers of innate inflammatory cells, e.g., monocytes, macrophages and neutrophils, to the spleen where blood filtration and parasite clearance takes place (28, 29).

We provide strong evidence that while both CD8^+^ and CD4^+^ T cells express LAG-3 at the time of anti-LAG-3 treatment (day 7.5), and represent the major LAG-3-expressing cells during *Py* infection (>80%), LAG-3 blockade is most likely acting on CD4^+^ T cells to unleash specific key effector mechanisms that help induce a more effective anti-parasite immune response. Despite an extensive analysis of these antigen-experienced parasite-specific CD4^+^ T cells across various time points post *Py* infection (day 7.5, 17 and 36), along with high dimensional 26 color flow cytometry panels, t-SNE and FlowSOM analysis of the CD4^+^ T cells in anti-LAG-3 or control isotype-treated PD-L1^-/-^ mice, we did not find any differences in CD4^+^ T cell subsets. The proportion of activated and proliferating CD4^+^ T cells, and of $T_{\text{bet}}^{+}\;(\text{Th}1)$, ICOS^+^, T_FH_ and T_FR_ remained equivalent in all experimental groups, consistent with the lack of detectable differences in the humoral response. Since cytokine production by the CD4^+^ T cells upon *in vitro* restimulation was the only functional read out that we found significantly different, we propose that LAG-3 blockade is likely to act as a rapid and temporary boost of activated CD4^+^ T cells in this infection. Perhaps akin to this experimental situation is the anti-PD-1 treatment in chronic virus-infected mice that re-invigorates exhausted CD8^+^ T cells, yet still fails to achieve long-term re-programming of these cells (30).

Even though our data support a functional role for CD4^+^ T cells in effective anti-parasite immunity, the fibrinogen-like protein 1 (FGL-1) appears as a major ligand of LAG-3 that works independently of MHC-II, and inhibits T cell-mediated anti-tumor immunity (4). While it is unknown whether levels of FGL-1 increase during malaria infection, an appealing hypothesis may be that FGL-1 contributes to inhibiting LAG-3 expressing subsets of T cells during infection, including CD8^+^ T cells. Along this hypothesis, we found a slightly delayed blood parasitemia clearance in anti-LAG-3-treated, CD8^+^ T cell-depleted PD-L1^-/-^ mice, and a mild increase in cytokines secreted after *ex vivo* stimulation of CD8^+^ T cells isolated from anti-LAG-3-treated *Py*-infected mice. Whereas we did find some modest differences, the lack of CD8^+^ T cells did not significantly affect the kinetics of blood parasitemia, consistent with prior studies using the same *Py17XNL1.1* strain and selective depletion or genetic depletion of CD8^+^ T cells (31–33). Of note, however, another group using a more virulent strain of *Py*, *Py NL*, documented a key role for CD8^+^ T cells in the control of blood parasitemia, invoking both IFNγ- and Fas-L-dependent protection mediated by the CD8^+^ T cells and macrophages (34, 35). The potential importance of CD8^+^ T cells during blood stage malaria, was also recently highlighted in *Py (17XNL1.1)*-infected transgenic mice expressing the human-restricted cytolytic effector molecule granulysin (GNLY) (36). Here, formal proof of concept that GNLY^+^ CD8^+^ T cells, shown to recognize and kill *P. vivax*-infected MHC-I^+^ reticulocytes (iRetics) *ex vivo* (37), could also kill MHC-I^+^ *Py-*iRetics in this model (36). Other studies using the *P. chabaudi* murine model of infection that induces low chronic blood-parasitemia rebounds over 1-3 months post infection (13, 38), have also reported CD8^+^ T cells, which express high cell-surface PD-1, to be essential in mediating effective blood stage parasite clearance during the chronic rebound phase and PD-1 blockade (13). Thus, while the above studies provide solid evidence supporting a role for CD8^+^ T cells during blood stage malaria, it is likely that this is accounted for by virulence features specific to the parasite strain such as its ability to infect reticulocytes (*P. vivax, P. yoelii*) and cause chronic episodes (*P. vivax, P. chabaudi*).

In the case of *Py 17XNL1.1* infections, however, all studies including ours, consistently report a more prominent role for CD4^+^ T cells in the control of blood stage parasitemia (8, 31, 32, 38). Thus LAG-3 blockade most likely acts through unmasking CD4 interactions with MHC-II to promote higher secretion of effector cytokines and -possibly- more robust recruitment/activation of innate effector cells. Even though we could only measure a limited impact of LAG-3/PD-L1 blockade on the immune response, the treatment significantly diminishes blood parasitemia (~30% from day ~12 and on), which may be essential in limiting the excessive inflammation associated with severe malaria. The treatment also appears to be effective at relatively high blood parasitemia, which furthers its potential therapeutic value. Overall, these inhibitory pathways appear to slow down host clearance of the parasite in this non-lethal animal model of malaria, but additional studies of these pathways in a lethal model, that better represents infection outcomes in humans, will be needed to understand the clinical implications of targeting these pathways during natural infection.

## Data Availability Statement

The original contributions presented in the study are included in the article/**Supplementary Material**. Further inquiries can be directed to the corresponding author.

## Ethics Statement

The animal study was reviewed and approved by The Institutional Animal Care and Use Committee of the Albert Einstein College of Medicine.

## Author Contributions

RF and LC designed, performed, and interpreted most experiments and contributed to figures and discussions. NZ and EG set up and conducted all Ab titer measurements. ES contributed to early experiments. SC contributed to the design of high dimensional flow cytometry panels. JD contributed to discussions and editing of the paper. GL designed experiments with RF and LC, assembled and edited figures and wrote the paper with critical reading by all authors. All authors contributed to the article and approved the submitted version.

## Funding

This work was funded by the National Institute of Health Grants (NIH/NIAID) AI103666 and AI128735 and Hirschl Caulier Award to GL. RF was supported by the NIH BETTR IRACDA training grant K12GM102779. LC received fellowships from Foundation Bettencourt-Schuller and the American Association of Immunology (AAI). ES and SC were supported by NIH training Grant T32A170117. EG was supported by NIH PREP R25GM104547.

## Conflict of Interest

The authors declare that the research was conducted in the absence of any commercial or financial relationships that could be construed as a potential conflict of interest.
