# Supplementary material for: Blockade of LAG-3 in PD-L1-Deficient Mice Enhances Clearance of Blood Stage Malaria Independent of Humoral Responses
Source: Front Immunol. 2021 Jan 14;11:576743. doi: 10.3389/fimmu.2020.576743 (PMC7840658; doi:10.3389/fimmu.2020.576743)
Supplement: Supplementary file 1 [file DataSheet_1.pdf]

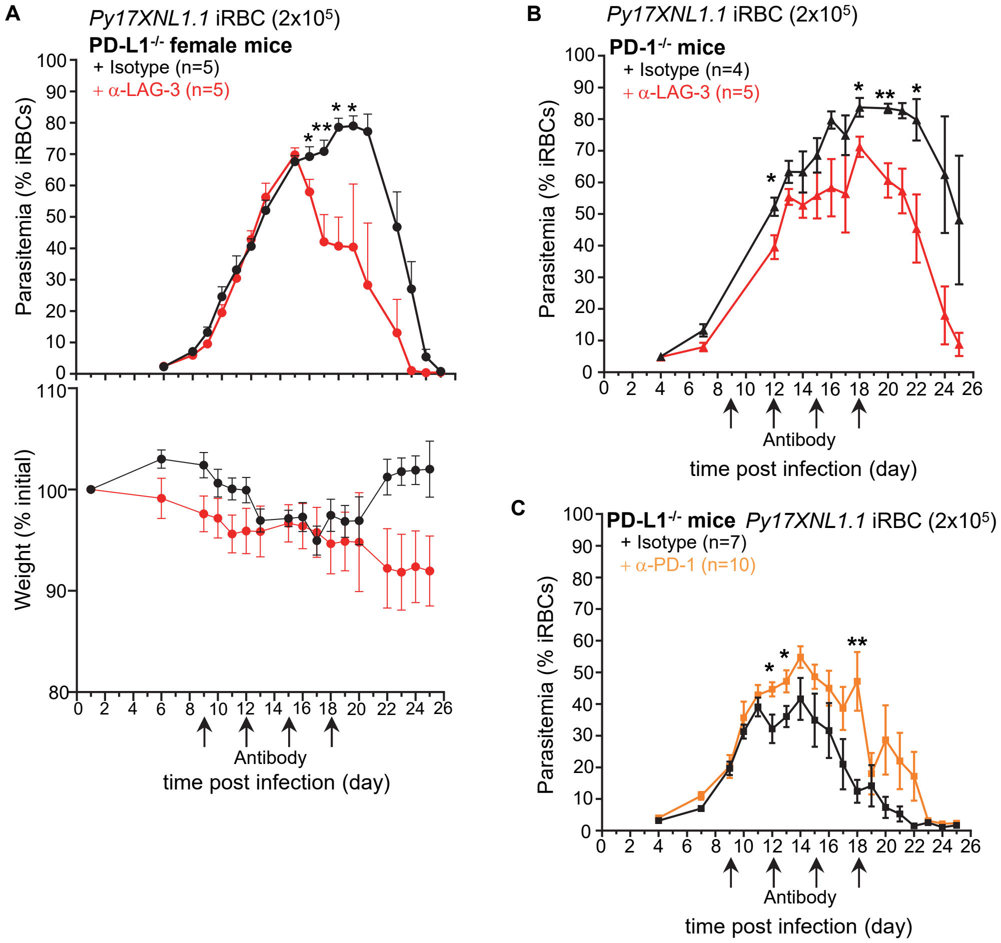

Supplementary Figure 1. LAG-3 blockade in female PD-L1$^{-/-}$ mice and PD-1$^{-/-}$ mice promotes reduced parasitemia. (A-C) Indicated genotype and gender mice were inoculated with 2x10^5 *Py* 17XNL1.1 iRBC i.v., and 9, 12, 15 and 18 days post infection, indicated mice received 300 mg (A) or 200 mg (B-C) of anti-LAG-3 or anti-PD-1 or isotype Ab i.v. Results show the kinetics of blood parasitemia over time, measured starting day 4 post infection and every day or every other day until day 25 using YOYO-1 staining of RBC and FACS. Graphs average the pool of 1-2 independent experiments shown with SEM (n=4-10). P-values are indicated when applicable (*p<0.05, **p<0.01) for each treatment compared to isotype group per time point by student's t-test.

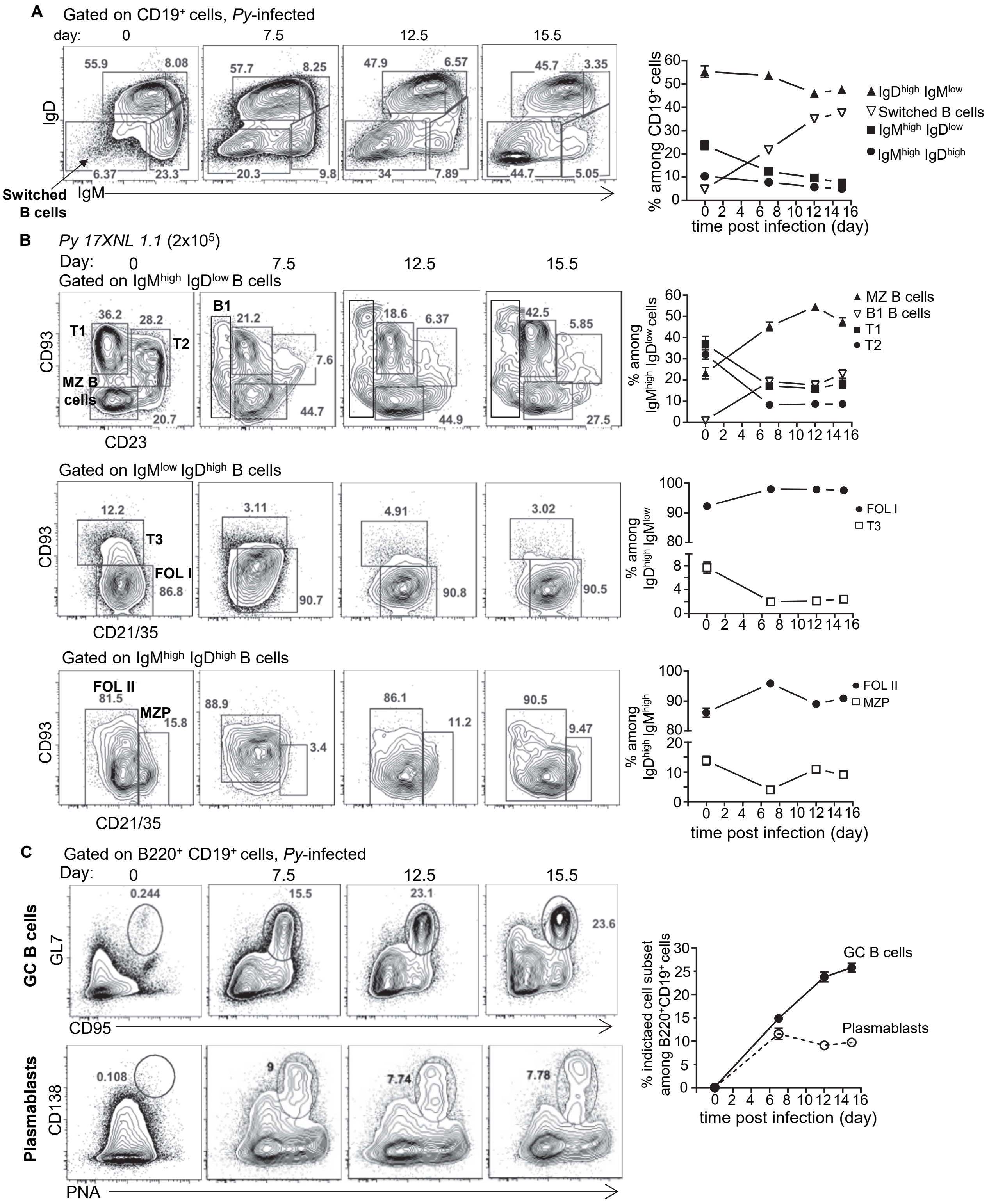

Supplementary Figure 2. Kinetic analysis of B cell responses in *Plasmodium yoelii*-infected mice. WT B6 mice were inoculated with 2x10^5 *Plasmodium yoelii (Py) 17XNL1.1* infected red blood cells (iRBC) i.v. **(A-C)** Spleens from *Py*-infected mice were harvested at indicated days post infection and cells stained with mAb against lineage markers (CD19, B220, CD3, CD4, Foxp3, Bcl6) and activation/subset markers (IgD, IgM, CD93, CD23, CD21/35, GL7, CD138, CD95, PNA). In all experiments, representative FACS dot plots of 3-5 independent replicate experiments are presented (n= 5-18). Graphs show average results from experiments with SEM.

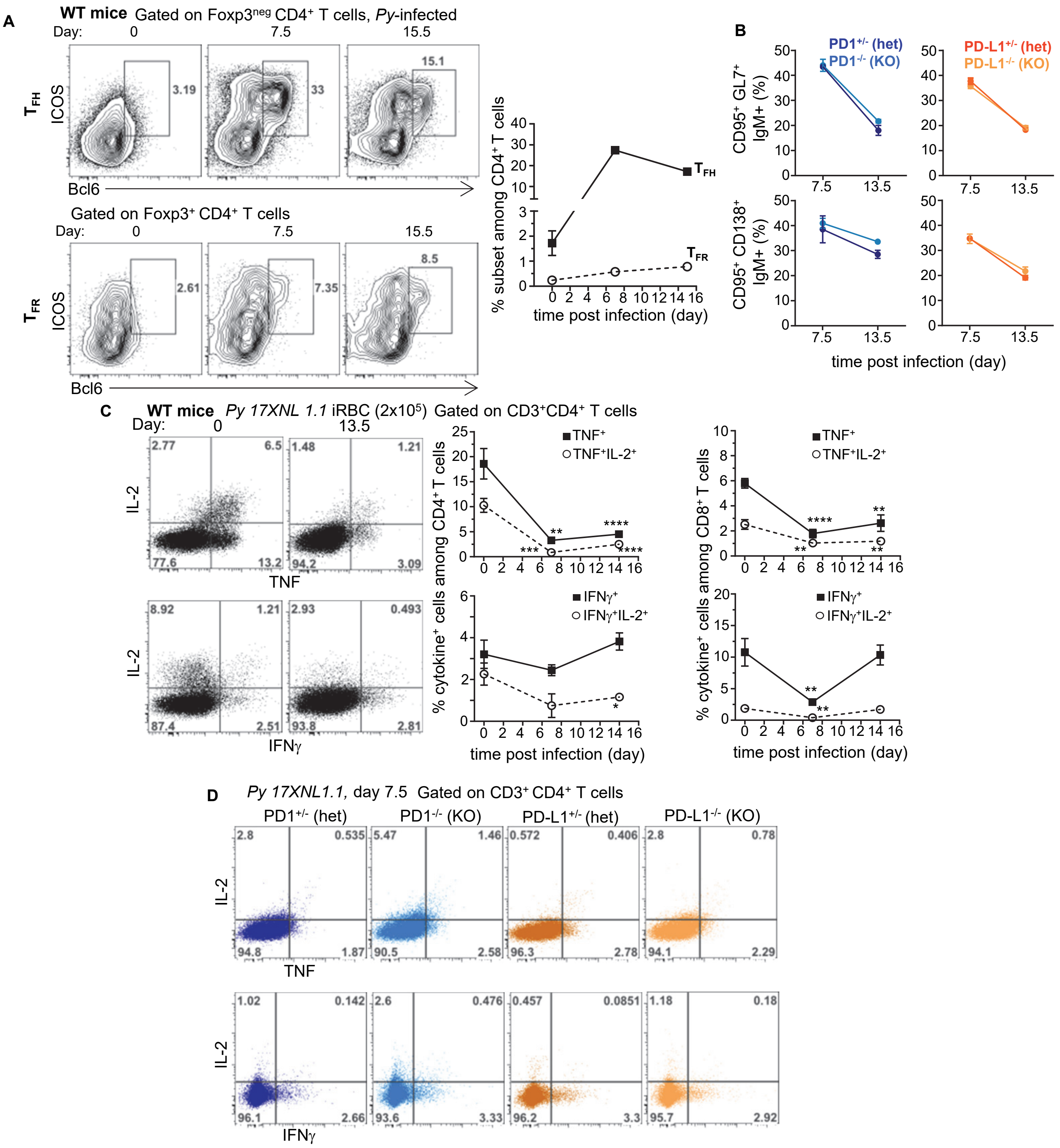

Supplementary Figure 3. CD4^{+} T cell responses in the spleen of *Py*-infected mice (A-D) Mice of indicated genotypes were inoculated with 2x10^{5} *Py* 17XNL1.1 iRBC i.v. **(A-B)** Spleens were harvested at indicated days post infection and cells stained with mAb against CD4, CD3, Foxp3, Bcl6, ICOS to monitor CD4^{+} T_{FH} and T_{FR} (A) or against lineage markers (CD45, CD3, CD11b,CD11c, CD19, Ly6C, B220) and activation/subset markers (IgD, IgM, CD93, CD23, CD21/35, GL7, CD138, CD95) (B). Representative FACS dot plots of 3-5 independent experiments are presented for 1 of 5-18 mice. Graphs present averages with SEM. **(C, D)** Spleen cells from day 7.5 *Py*-infected WT (C) or littermates mice of indicated genotypes (D) were incubated with PMA/ionomycin for 4 hours and stained for cell-surface CD3 and CD4 and intracellular cytokines IL-2, IFNγ and TNF. Cytokine-producing cell frequencies among CD4^{+} T cells are shown in a representative FACS dot plot of 2 independent experiments (n= 3-12). Student's t-test was done and p-values reported when applicable (*p<0.05,**p<0.01,***p<0.001)

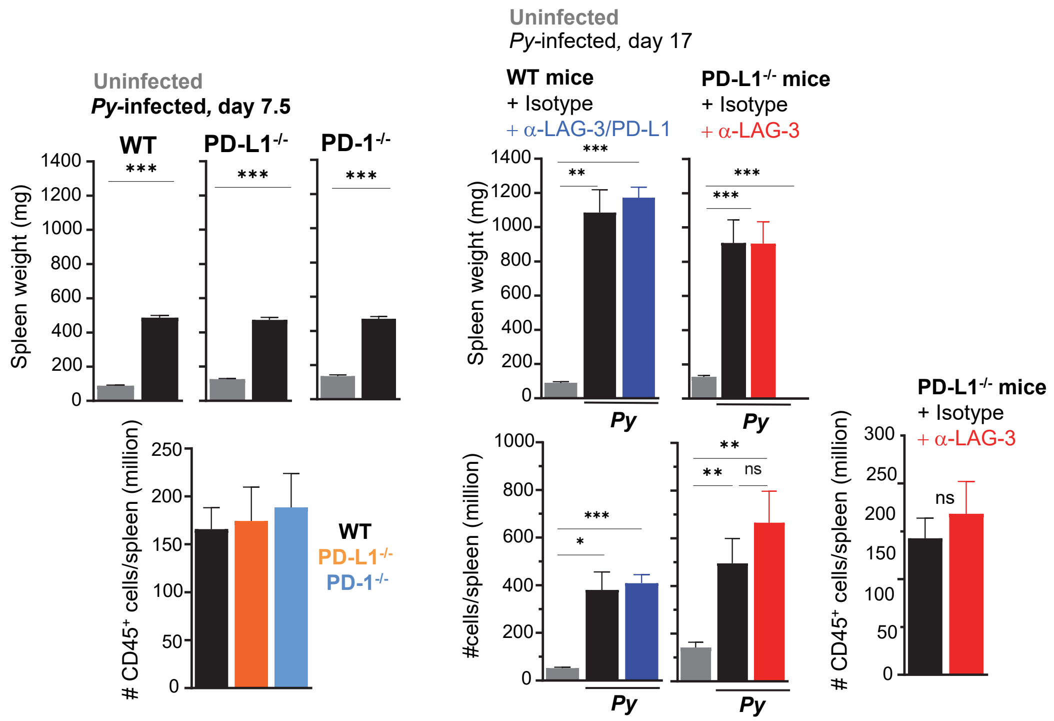

Supplementary Figure 4. Increase in spleen size and spleen cell numbers in *Py*-infected compared to uninfected mice. Spleen weight, total and hematopoietic ($CD45^+$) cell numbers in uninfected or *Py*-infected ($2x10^5$ *Py* 17XNL1.1 iRBC i.v) in WT, PD-L1$^{-/-}$, PD-1$^{-/-}$ mice on day 7.5 post *Py*-infection (n=3-5 mice) or in WT or PD-L1$^{-/-}$ mice treated (day 9, 12, 15) with anti-LAG-3/PD-L1, anti-LAG-3 or matching isotype 17 days post *Py*-infection pooled across 2 experiments (n=4-10 mice). Student's t-test was conducted and p-values reported as indicated between groups when applicable (*p<0.05, **p<0.01, ***p<0.001).

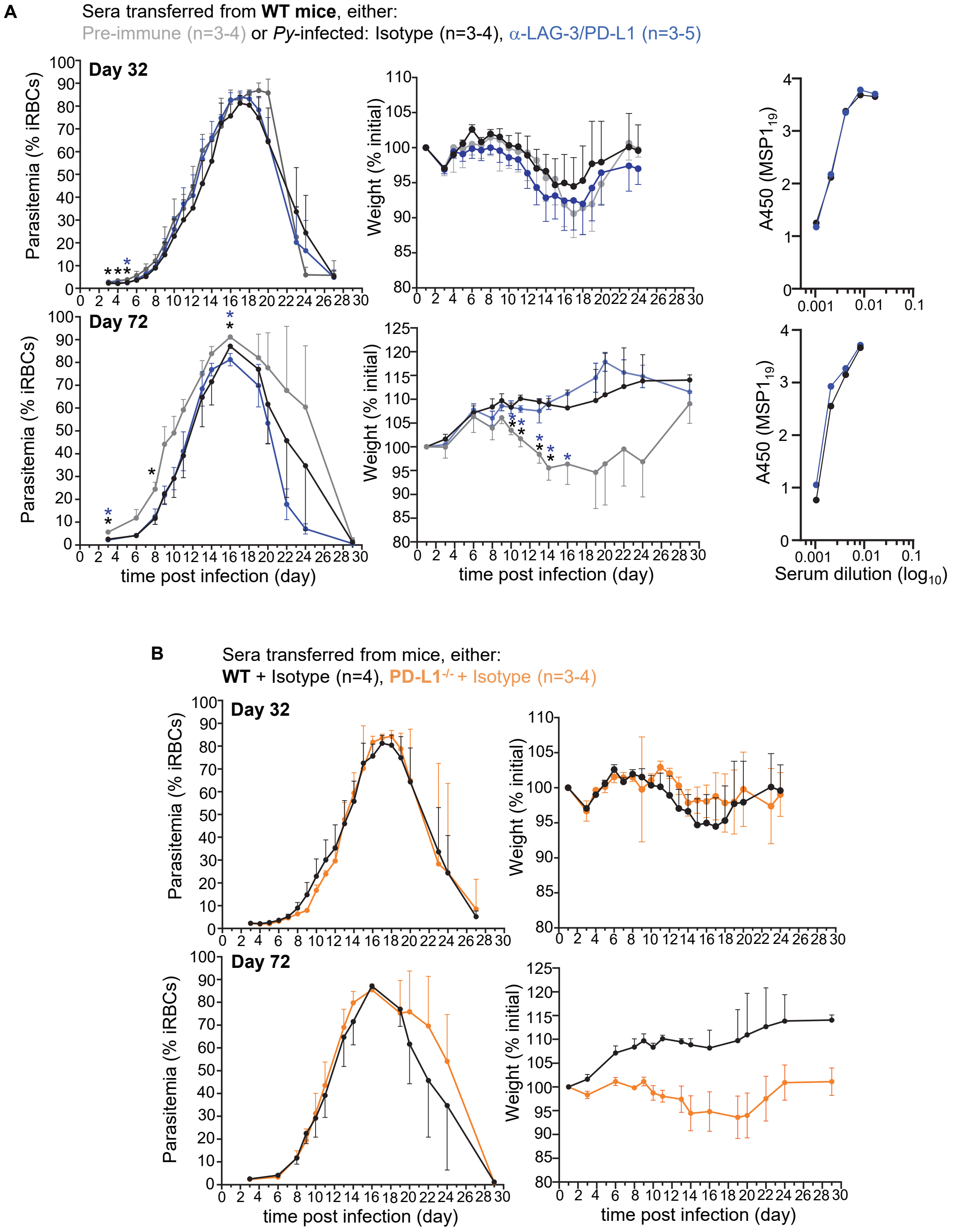

Supplementary Figure 5. LAG-3 neutralization in WT mice fails to promote the production of higher titers and more protective parasite-specific antibodies. Blood parasitemia and change in initial weight of WT mice measured over time starting day 3 post inoculation with $2x10^5$ *Py* 17XNL1.1 iRBC i.v. Prior to infection, mice received 150 µl of sera from **(A)** either pre-immune WT mice or sera collected from isotype or anti-LAG-3/PD-L1 treated *Py*-infected WT on either day 32 or 72 post infection. Titers of $MSP_{1\text{-}19}$–specific IgG Abs in indicated sera detected by ELISA as described in the methods. In **(B)** sera comes from either isotype treated *Py*-infected WT or PD-L1$^{-/-}$ mice at day 32 or 72.

A **26 color Cytek Panel**

Lineage Markers	Transcription Factors	Chemokine Receptors	Functional Markers		Antigen Experience
CD45 AF700<br>CD3 BV750<br>CD4 PerCP-Cy5.5 (day 7.5)<br>CD4 PE-Cy7 (day 36)<br>CD8 AF532	BCL6 APC-Cy7<br>EOMES eF660<br>FOXP3 AF488 (day 7.5)<br>TCF1 AF488 (day 36)<br>Ki67 PE-Cy5 (day 7.5)<br>Ki67 eF450 (day 36)<br>Tbet BV711	CCR5 PerCP-eF710 (day 7.5)<br>CCR7 BV786<br>CXCR3 PerCP-Cy5.5 (day 7.5)<br>CXCR5 BUV661 (day 7.5)<br>CX3CR1 BV510	CD27 BV650<br>CD44 SB436<br>CD62L BV570<br>CD95 BB515<br>CD122-BUV496<br>CD127-BV421	Granzyme B APC<br>ICOS PE-Cy7 (day 7.5)<br>KLRG1 BV605<br>PD1 BUV737<br>Sca-1 BUV395	CD11a PerCP-eF710 (day 7.5)<br>CD11a PE (day 36)<br>CD49d PE (day 7.5)

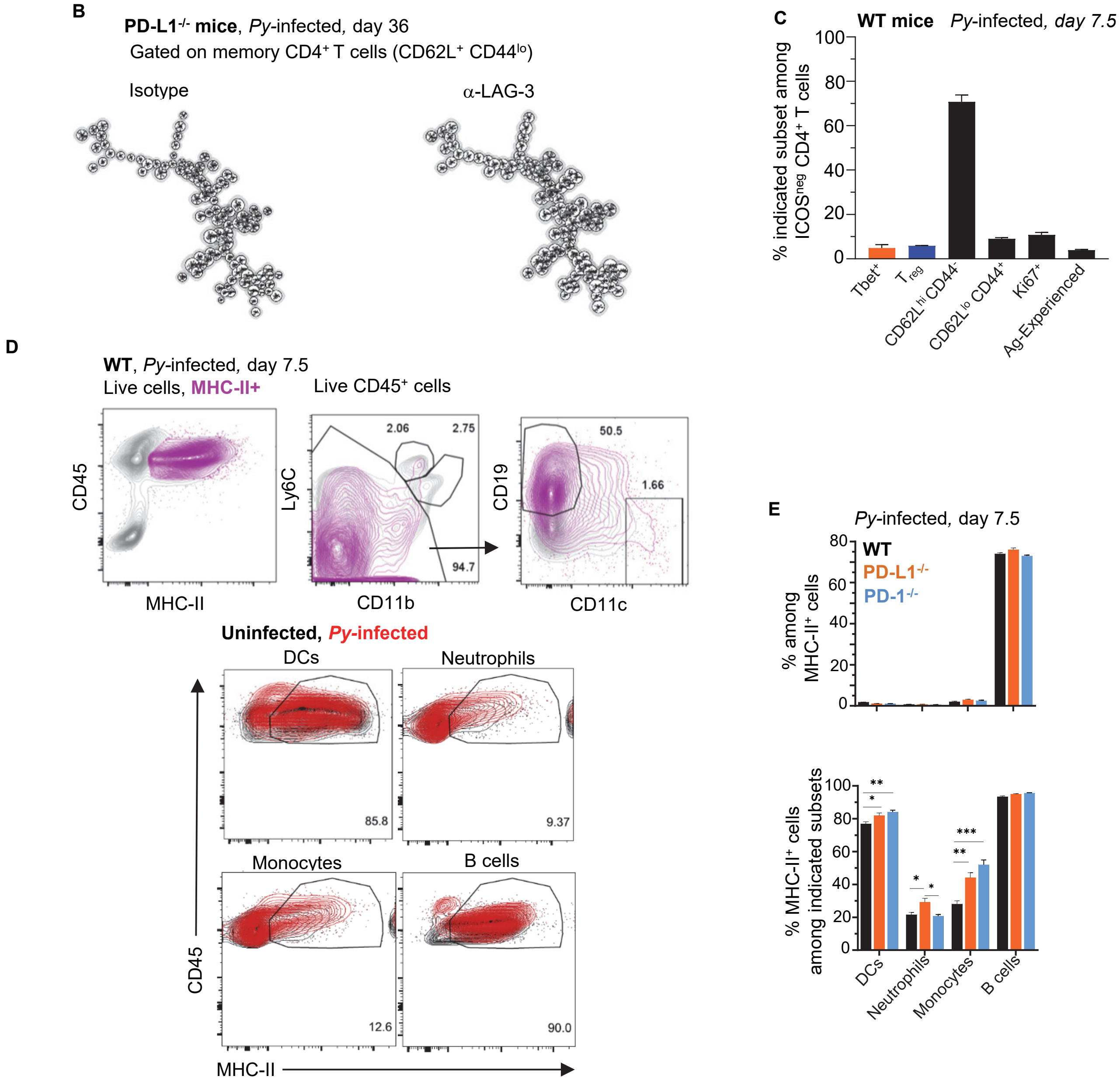

Supplementary Figure 6. High dimensional flow cytometry panel to characterize CD4$^+$ and CD8$^+$ T cells in *Py*-infected mice. (A) Panel of Abs applied to splenocytes from WT mice at either day 7.5 or day 36 post *Py 17XNL1.1* infection, as indicated, for staining and analysis on the Cytek Aurora cytometer. **(B)** Spleen cells from day 36 PD-L1$^{-/-}$ *Py*-infected mice treated with either anti-LAG-3 or isotype Ab i.v. (on days 9, 12, 15) were stained for lineage markers (CD8, CD4, CD3, Bcl6, T$_{bet}$) and functional markers (CD62L, CD44, CD11a) with a 24 color panel depicted in (A). FlowSOM analysis on pooled (n=4-5) memory (CD62L$^+$ CD44^{lo}) CD4$^+$ T cells are presented. **(C)** Indicated cell subset frequencies among ICOS negative splenocytes in WT mice on day 7.5 post *Py* infection. Bar graphs show average cell frequencies with SEM. **(D, E)** Indicated cell subset frequencies among spleen cells in WT (D) or PD-1$^{-/-}$ or PD-L1$^{-/-}$ (E) mice (n=4-5 mice per group) on day 7.5 post *Py* infection stained with mAb against lineage markers (CD45, CD19, CD3, CD4) and activation/subset markers (MHC-II, CD11c, CD11b, Ly6C). Student's t-test was conducted and p-values reported as indicated between groups (*p<0.05, **p<0.01, ***p<0.001).
