# Supplementary material for: Blockade of LAG-3 in PD-L1-Deficient Mice Enhances Clearance of Blood Stage Malaria Independent of Humoral Responses
Source: Front Immunol. 2021 Jan 14;11:576743. doi: 10.3389/fimmu.2020.576743 (PMC7840658; doi:10.3389/fimmu.2020.576743)
Supplement: Supplementary file 2 [file DataSheet_2.pdf]

Supplementary Table 1. List of antibodies used for flow cytometry analyses and in vivo blocking or cell depletion.

Reagent/Antibody Target	Fluorochrome	Clone	Purchased from
**FACS**			
anti-rat	biotin	Polyclonal (Goat)	Invitrogen
B220	PE-Cy7	RA3-6B2	eBiosciences
BCL6	PE, APC-Cy7	K112-91	BD Biosciences
CCR5	PerCP-eF710	7A4	Invitrogen
CCR7 (CD197)	BV786	4B12	BD Biosciences
CD115	PE-Cy7	AFS98	eBiosciences
CD11a	APC, PE, PE-Cy7, PerCP-eF710	M15/4, M17/4	eBiosciences, Biolegend
CD11b	BB700, PerCP-Cy5.5	M1/70	BD Pharmingen
CD11c	biotin, APC	HL3	BD Pharmingen
CD122 (IL-2Rb)	BUV496	TM-b1	BD Biosciences
CD127 (IL-7Ra)	BV421	A7R34	BD Biosciences
CD138	PE	281-2	Biolegend
CD169	PE	SER-4	eBiosciences
CD19	AF700, PerCP-Cy5.5	1D3	BD Pharmingen
CD27	BV650	LG.3A10	BD Biosciences
CD3	BV510, FITC, PerCP-Cy5.5, PE-Cy7, BV750	145-2C11 or 17A2	eBiosciences, BD Biosciences
CD4	Pacific Blue, PE, Alexa 700, PE-Cy7, PerCP-Cy5.5	RM4-5	eBiosciences, BD Pharmingen, Biolegend
CD44	SB436	IM7	eBiosciences
CD45	Alexa 700, APC	30F11	eBiosciences
CD49d	PE	R1-2	eBiosciences
CD62L	BV570	MEL-14	BioLegend
CD8a	FITC, PE, PerCP.Cy5.5, APC, Alexa 700, BV510, PE-Cy7, AF532	53-6.7	eBiosciences
CD95	FITC, BB515	Jo2	BD Biosciences
CX3CR1	BV510	SA011F11	BioLegend
CXCR3	PerCP.Cy5.5	CXCR3-173	BioLegend
CXCR5	unconjugated, PE-Cy7	2G8	BD Biosciences
EOMES	eFluor660	Dan11mag	eBiosciences
F4/80	BV421	BM8	BioLegend
FoxP3	eF450, AF488	FJK165, FJK-16s	eBiosciences
GL7	eF450	GL7	eBiosciences
Granzyme B	APC	MHGB05	Invitrogen
ICAM1	PE	3E2	BD Biosciences
ICOS	APC, PerCP-eF710, PE-Cy7	C398.4A, 15F9	eBiosciences
IFNg	AF700, PE-Cy7	XMG1.2	eBiosciences
IgD	PerCP-Cy5.5	11-26c.2a	BD Pharmingen
IgG	AF700	Poly4053	Biolegend
IgM	APC	11-41	eBiosciences
IL-2	eFluor 450	JES6-5H4	eBiosciences
Ki67	FITC, eF450, PE-Cy5	SolA15	eBiosciences
KLRG1	BV605	2F1	BD Biosciences
LAG3	APC	C9BTW	BD Pharmingen
Ly6C	AF700	AL-21	BD Pharmingen
Ly6G	FITC	1A8	BD Biosciences
MHCII	PE-Cy7	M5/114.15.2	Biolegend
PD1	PE, FITC, BUV737	J43, RPM1-30	BD Biosciences, eBiosciences
PNA	FITC		Sigma-Aldrich
SCA-1 (Ly6A/E)	BUV395	D7	BD Biosciences
Streptavidin	BUV661, APC-Cy7, PE-Cy7		eBiosciences, BD Biosciences
Tbet	BV711	4B10	Biolegend
TCF1	AF488	812145	R&D
TNFa	PerCP-Cy5.5	MP6-XT22	BD Biosciences
**In vivo depletion antibody**			
anti-CD4		GK1.5	
anti-CD8b		H35	
**In vivo blocking antibody**			
polyclonal Rat IgG		N/A	BioXcell
anti-LAG3		C9B7W	BioXcell
anti-PD-1		RMP1-14	BioXcell
anti-PD-L1		10F.9G2	BioXcell
